# Navigating sexual and reproductive health among Venezuelan women sex workers in Peru: Qualitative explorations of structural vulnerability and resistance

**DOI:** 10.1371/journal.pgph.0006906

**Published:** 2026-07-27

**Authors:** Mariangela Castro-Arteaga, Julien Brisson, Juan Carlos Ramírez Tovar, Karla Solari Perez, Deana Franceska León Morris, Dorothy Apedaile, Kelika Konda, Alfonso Silva-Santisteban, Amaya Perez-Brumer

**Affiliations:** 1 Department of Geography and Planning, University of Toronto, Toronto, Ontario, Canada; 2 Dalla Lana School of Public Health, University of Toronto, Toronto, Ontario, Canada; 3 Faculty of Pharmacy, University of Montreal, Montreal, Quebec, Canada; 4 Unit of Health, Sexuality, and Human Development, Universidad Peruana Cayetano Heredia, Lima, Peru; 5 Keck School of Medicine, University of Southern California, Los Angeles, California, United States of America; PLOS: Public Library of Science, UNITED STATES OF AMERICA

## Abstract

Migration to Peru from Venezuela has reshaped engagement in informal labour for many women who turn to sex work as a survival strategy amid legal precarity and systemic exclusion. Yet little is known about how migrant sex workers navigate sexual and reproductive health (SRH) in this context. In 2023, we conducted 34 semi-structured interviews with Venezuelan migrant cisgender women sex workers living in Lima, Peru. Interviews explored migration trajectories, working conditions, SRH needs, and experiences seeking care. Analysis was guided by the Structural Vulnerability Framework, contextualizing how institutional framings and public health logics structure SRH access, exclusion, and women’s adaptive strategies. Participants described access to SRH services in Peru as narrowly restricted to HIV and sexually transmitted infection (STI) testing and condom distribution, with accessibility to those services often contingent upon disclosing involvement in sex work, thereby reinforcing surveillance, stigma, and experiences of mistreatment in healthcare settings. Comprehensive SRH needs—including contraception, cervical and breast cancer screening, management of menstrual disorders, and responses to condom breakage or occupational injuries—were frequently unaffordable, unavailable, or dismissed, even as these conditions directly affected women’s capacity to work. Responding to structural gaps in care, participants exercised agency within constrained landscapes, organizing peer networks and relying on community-based organizations to access SRH information, emotional support, and care. The normalization of out-of-pocket payments further illustrated how health decisions were made within constrained economic and bureaucratic contexts. Urgent and structural action is needed to close bureaucratic barriers to access to health insurance for migrants, implement non-stigmatizing models of care, and support partnerships with peer networks and community organizations.

## Introduction

Latin America has experienced its largest population displacements in recent history due to Venezuela’s political, economic, and social turmoil, which intensified in 2017 [1,2]. Peru has become a primary destination, hosting around 1.7 million Venezuelan migrants by early 2025 [3], with women accounting for 50.6% of this migrant population [4]. Notably, Lima is home to the largest number of Venezuelans outside Venezuela [5,6], with 80% of Venezuelans in Peru concentrated in the city [7]. In Peru, where approximately 75% of the total workforce is engaged in informal employment [8,9], Venezuelan migrant women are further segregated into gendered sectors such as domestic work and general services [10–12]. For many, however, sex work emerges as a survival strategy post-migration in the face of economic precarity and social exclusion [13–16]. This context exposes them to heightened risks of violence, negative health outcomes, sexual and labour exploitation, and human trafficking [17–19]. Cisgender Venezuelan women in Peru are subjected to hypersexualized stereotypes and xenophobic attitudes [20,21], and face an even greater burden of chronic health conditions and unmet sexual and reproductive health (SRH) needs. For instance, only 20.6% of Venezuelan women received family planning services, compared to 56% of the general population in Peru [22]. Against this complex backdrop, this study examines how Venezuelan migrant cisgender women engaged in sex work in Lima navigate SRH services, with attention to how migration, gender, and labour intersect to shape SRH vulnerabilities and access to care.

In Peru, sex work is legal but unregulated, a situation that excludes sex workers from labour protections, social safeguards, and institutional support, leading to precarious and exploitative working practices [16]. Within the public health system, sex workers are not formally recognized as workers, instead they are classified as a “high-risk group” and are primarily approached through a disease-prevention lens in relation to sexually transmitted infections (STIs), including HIV [23]. This framing underpins the Ministry of Health’s guidelines [24] mandating quarterly HIV/STI testing and condom provision, alongside hepatitis B/C screening and vaccination for sex workers regardless of migration status and health insurance coverage. Other SRH needs, such as gynecological health (e.g., menstrual disorders, pelvic health/dysfunction), safe perinatal care, prevention of gender-based violence, HPV-related cancer prevention, and the right to access SRH information and education [25–27], are only accessible to those with health insurance or who can pay. Public health insurance (*Sistema Integral de Salud SIS)* eligibility is limited to individuals with Peruvian citizenship or a foreign resident card (*carnet de extranjería*) [28], which demands additional payments and navigating a bureaucratic process that is often unattainable for migrant sex workers [29].

Peru’s constitution guarantees the right to health for all [30], while more recent legislation has recognized that foreign nationals have the same fundamental rights as Peruvian citizens [31]. Universal health insurance is nominally available to all those residing in Peru (Law No. 26842 [32] and Universal Health Insurance Law 29344 [33]), while the Sexual and Reproductive Health Law (2010) similarly defines SRH as a human right, emphasizing gender equity, universal access, and community participation [34]. Taken together, these legal frameworks suggest that Venezuelan migrant sex workers should, at least on paper, be doubly protected: as migrants formally included within universal health guarantees and as individuals entitled to non-discriminatory SRH care irrespective of occupation. However, previous studies have shown that Venezuelan migrant women are subject to heightened discrimination in healthcare settings, including SRH care services [23,35,36]. Furthermore, while SIS eligibility has been extended to individuals up to 42 days postpartum regardless of migration status and health insurance [37,38], barriers persist for migrant sex workers who are unable to retain SIS eligibility.

To explore the SRH needs of Venezuelan migrant women who engage in sex work, this study draws on the Structural Vulnerability Framework [39–42]. The Structural Vulnerability Framework conceptualizes health outcomes as products of political, economic, and social arrangements rather than individual behaviors, emphasizing how intersecting systems of inequality, such as migration status and gender, produce patterned exposure to harm. It also serves as a practical lens for identifying and addressing these structural barriers through systems-level interventions. Prior research applying this framework has demonstrated how legal migratory barriers and internalized marginalization produce healthcare exclusion and shape agency among street-based women in Nigeria [43], how short-term mobility and structural constraints amplify violence, sexual risk, and barriers to care among sex workers [42], and how migration broadly reorganizes access to care through political and economic stratification [39].

In Peru, Venezuelan migrant sex workers occupy a structurally vulnerable position at the intersection of precarious migration status, unregulated and stigmatized labour, and exclusion from social protection schemes such as SIS. Structural vulnerability helps illuminate how these interlocking systems constrain SRH access and occupational health, while also shaping the strategies of resistance and self-care that women develop in response, including paying out-of-pocket, peer knowledge exchange, and reliance on community-based organizations (CBOs) or non-governmental organizations (NGOs). The framework resists purely victimizing accounts by foregrounding agency within constraint and offering a critical lens for identifying where health inequities are produced and where structural, rather than solely individual-level, interventions are required to advance SRH equity.

This paper explores how Venezuelan migrant sex workers navigate access and structural barriers to SRH and occupational health services, and examines the roles played by peer networks, CBOs and NGOs in facilitating SRH care. By centering their experiences, this paper contributes to ongoing debates on health equity, sexual rights, and advances the need for comprehensive, rights-based SRH for migrant sex workers.

## Methods

### Ethics Statement

Proyecto RADIANTE obtained approval from the University of Toronto’s Research Ethics Board #43980 and the Universidad Peruana Cayetano Heredia in Peru # 210661. Participants provided written informed consent prior to participation and were given a list of free resources to contact if needed. Participants received 50 Peruvian Soles (~14USD) for their participation.

Participants were assigned pseudonyms to protect their identities. Audio recordings and transcripts were uploaded to a password - protected server accessible only to select team members. As per institutional policies, the data will be retained for 7 years after data collection is completed, then destroyed.

### Study design

“Proyecto RADIANTE: Enhancing HIV, sexually transmitted, and blood-borne infection (STBBI) prevention and care among refugee and displaced Venezuelans engaged in sex work in Lima, Peru”, is a sequential exploratory mixed-methods study that included individual semi-structured interviews with Venezuelan migrant sex workers, which informed the development of a biobehavioral cross-sectional survey with the same population. This paper focuses on findings from the semi-structured interviews with Venezuelan migrant cisgender women who engage in sex work. Semi-structured interviews offered participants the opportunity to reflect on and elaborate on their experiences, particularly regarding complex and sensitive topics such as the intersection of migration, sex work, and health.

### Participants and data collection

Interviews were conducted between August 23, 2023, and December 14, 2023. Participants had to be at least 18 years old, identify as cisgender women (self-identify as women with a female sex assigned at birth), able to provide informed consent, self-identify as Venezuelan, migrated to Peru within the past 5 years (i.e., since 2018), self-describe as a sex worker or as someone who engages in sex work, and reside in Lima, Peru. Recruitment was conducted in collaboration with ‘Asociación de Trabajadoras Sexuales Miluska Vida y Dignidad’, a community-based organization (CBO) with a strong presence in Peru that advocates for social inclusion and health rights of sex workers. CBO members, which included Peruvian and Venezuelan migrant cisgender women sex workers, distributed flyers with study information in settings where sex workers were present and leveraged pre-existing connections between the organization and potential participants. Additionally, enrolled participants circulated information about the study and invited other eligible Venezuelan women residing in Lima through snowball sampling.

This study focused specifically on cisgender women, given the convergence of intersecting structural conditions shaping their engagement with sex work and access to SRH services in Peru and their specific sex- and gender-based SRH needs [44,45]

Sample size was determined by information power and supported by ongoing assessment of thematic sufficiency [46], which allowed for adjusting the sample based on the relevance of the obtained information and for identifying key patterns among participants as data collection progressed. Given the study’s focused objective on SRH among Venezuelan migrant women engaged in sex work in Lima, the specificity of the sample, and the depth of the in-depth interviews, we determined that a sample of 34 participants provided sufficient informational richness to support robust analysis. This assessment was supported by iterative team discussions during data collection and preliminary analysis, which indicated that additional interviews were yielding limited novel insights relevant to the study objectives. Accordingly, the findings reflect participants’ situated experiences and interpretations rather than an exhaustive account of all dimensions of SRH access. As a data collection method, the team developed a semi-structured interview guide that touched on the following core themes: exploring migration trajectories, SRH needs during relocation, perceptions of sex work, and HIV/STI care. The research team, comprised of native Spanish-speaking qualitative researchers, conducted interviews in person, lasting approximately 60–90 minutes in a private space.

Interviews were conducted by three cisgender women researchers (two Peruvian and one Venezuelan) who had formal training in qualitative methods and prior experience working with sex worker communities. The decision to have cisgender women conduct the interviews was intentional, with the aim of fostering rapport and facilitating open discussion of highly sensitive topics related to sex work, migration, and sexual and reproductive health. At the same time, we recognize that shared gender identity does not eliminate power differentials, and that researchers’ social positionalities may have shaped both what participants chose to disclose and how experiences were narrated. We remained attentive to how cultural and national origins, as well as institutional affiliation, may have influenced participants’ expectations of the research and the ways in which they framed their narratives. All interviews were conducted in private settings to promote confidentiality and participant comfort. Prior to each interview, researchers emphasized the voluntary nature of participation, the confidentiality of responses, and the study’s purpose of creating space for participants to share their perspectives, with the broader goal of informing advocacy and policy measures responsive to their needs.

### Data analysis

Interviews were transcribed verbatim and coded in Spanish using Dedoose Version 7.0.23 to preserve the conceptual integrity of participants’ accounts and reflect the cultural and linguistic contexts in which their experiences were situated [47]. Intra- and inter-coder consistency was ensured through team-based coding, cross-coding of 10% of the data, and regular discussions to address analytic tensions and reconcile discrepancies. The data analysis followed an immersion-crystallization approach, in which data collection and analysis were integrated in parallel through an iterative and reflexive process, enabling the identification of meaningful themes [48]. Guided by the Structural Vulnerability Framework, the research team approached analysis with a focus on how healthcare precarity, institutional neglect, and intersecting forms of stigma (e.g., xenophobia, anti-Venezuelan sentiment) were embedded in participants’ narratives shaping systems of exclusion. Coding and theme development were informed by this framework accounting not only for participants’ SRH encounters but also on how these encounters were mediated by legal status, gendered labour hierarchies, and access to care. Attention was also placed on how participants exercised agency within constraint, for instance, by strategically engaging with informal care networks, building peer-led knowledge-sharing practices, and/or resisting stigmatizing clinical environments. Throughout analysis, the research team engaged in ongoing reflexive dialogue, documenting analytic decisions and interrogating how our disciplinary training, prior advocacy engagements, and positionalities as researchers working in migration and SRH may have shaped interpretation of the data. Reflexive memos were used to critically examine assumptions, emotional responses, and potential normative commitments, particularly regarding sex work, stigma, and structural injustice, to mitigate overdetermined readings of participants’ accounts. Only quotes selected for publication were translated into English, with careful attention to preserving tone, meaning, and affect.

## Results

A total of 34 Venezuelan cisgender women participated in the interviews, with a mean age of 33 years (range: 21–52). The majority (88%; n = 30) were not registered with Peru’s public health system (*Sistema Integrado de Salud* – SIS). Nearly all participants (n = 33) were mothers, and most (n = 24) reported initiating sex work after migrating to Peru. Drawing on the Structural Vulnerability Framework, participants’ experiences are analyzed and presented below as shaped by structural forces, including migration policy, labour precarity, and gendered stigma that constrain access to care and produce patterned inequalities in SRH outcomes. The three interrelated themes identified include: 1. Conditional care shaped by structural factors including institutional policies, migration status, and stigmatized forms of labour that structure availability and the terms under which SRH is accessed; 2. Occupational health access constrained by economic and bureaucratic contexts, shaped by out-of-pocket expenditures, insurance eligibility and institutional responses to stigmatized labour; and 3. Community-based care in response to structural exclusion and conditional access, exercising agency within structural constraints.

### 1. Conditional care and intersectional stigma in SRH access post-migration

Participants emphasized the importance of accessing comprehensive SRH services for their health and wellbeing. However, they described this access as limited to HIV/STI testing and condom distribution services contingent upon disclosure of engagement in sex work, reinforcing dynamics of both surveillance and discrimination. As Sofia (39 years old) explained: “You have to tell the nurse: ‘I’m a sex worker and I’m here to get tested’. If you don’t get HIV/STI testing, they don’t give you condoms.” This conditional access rendered sex workers hypervisible in clinical encounters, an environment permeated by moral judgment and mistreatment by providers within the public clinics. As Valentina (27 years old) also shared, “Everything is fine until you say you’re a sex worker. They see you as a dirty person, an indecent person, a worthless person.”

Given the layers of structural and interpersonal stigma related to migration and sex work, many participants chose to avoid the public healthcare system altogether despite having SIS insurance. As Valeria (40 years old) explained:

Most of them [doctors] see us as weirdos, first because we are Venezuelan, and if they know about the sex work, they treat you even worse. I prefer to go private clinics because at the *posta* [i.e., publicly funded clinic] they treat me badly. When they do a Pap smear, I tell the healthcare staff that they are hurting me and they say ‘you have to hold on’. It is a total mistreatment. In a private clinic, they talk to you nicely and you don’t feel mistreated in any way.

While HIV/STI testing is provided contingent upon occupation disclosure, broader SRH needs, like preventive care (e.g., HPV vaccination, cancer screening), counseling (e.g., mental health services, prevention of gender-based violence), or treatment for chronic issues (e.g., menstrual disorders), remained unaddressed and unaffordable for participants. As shown below in Valentina’s (27 years old) quote, participants actively identify their SRH needs in relation to their work, yet must pursue care within structurally constrained conditions that limit access to comprehensive services:

If you are a sex worker, there’s a special section where you go for tests and that’s it. I mean, they test you for HIV, syphilis, and other infections but they don’t offer more than that. Now, without SIS, if we have a headache, cancer or we need an ultrasound, we can’t access any of those services, we have to pay for everything. We only receive our monthly box of condoms and an HIV test, and that’s it, period.

Facing these barriers, participants without SIS insurance who could afford payments described supplementing services through out-of-pocket payments for additional SRH care. As Bianca, (28 years old) explained, “I get tested for HIV regularly because of the job, but I have to pay out of pocket for Pap smears. Sometimes I don’t have the money, but it’s something I have to do.” Similarly, Giselle (25 years old), who does not have health insurance, noted: “I often go to a private clinic for transvaginal ultrasounds to make sure I’m okay, you know? I get Pap smears and all that. I go every five or six months, but I see the gynecologist every two months because I know the kind of work I do.” These accounts highlight how participants identify their SRH needs based on their sex work practices as well as the constrained conditions under which SRH care must be pursued, even when doing so caused personal financial strain.

In terms of reproductive care, participants without SIS insurance shared similar experiences of paying out-of-pocket for contraceptives: “I paid for the birth control implant; as long as I pay, I do not have any issues” (Isabel, 25 years old). Additionally, the reduction of sex workers’ SRH needs to condom distribution has restricted access to more comprehensive information and reproductive services. When asked if she had accessed any reproductive care in Peru, Beatriz (35 years old) stated that she had never used these services in the country because she did not know “where they provide such services”. She added: “Obviously, I have my checkups with my private gynecologist for HIV care, but not for reproductive care.” In other cases, family planning information was accessed through student-led initiatives or NGOs, but only as a pathway to condom distribution. For example, Carolina’s (42 years old) account illustrates that condoms are distributed as a “work tool” while other methods are lacking. She shared: “I don’t have any regular contraceptive method even though I’m sex worker. Once we participate in those family planning informational sessions they distribute condoms, which is our work tool.”

### 2. Sex work and occupational SRH needs

Sex workers routinely face occupational hazards that directly threaten their health, with condom breakages representing a particularly common risk that can expose them to STIs and unwanted pregnancies. These incidents can occur unintentionally or intentionally (e.g., through client sabotage). When seeking medical care after these urgent situations, participants reported experiencing hostile interactions such as intimidation, disrespect, and dismissive attitudes by healthcare center staff, in addition to substandard clinical services within the public health system. As explained by Estefania (38 years old):

One time, a client’s condom broke, and I was scared. When I went to the public health center, the guard at the entrance did not want to let me in. He even started yelling at me. He treated me like garbage. I got checked anyways, but it wasn’t what I expected. I wanted them to properly check me and do some testing. But no. I went [to a private clinic] and had the tests, pap smear and everything and I had to pay for everything. If they’re going to treat me badly, I’d rather pay for my own things, and besides, the private clinic gives me the results faster.

Structural vulnerability was described as operating through both institutional responses and economic precarity, shaping not only exposure to risk but also access to timely and adequate care. Indeed, the physical demands of sex work exacerbated chronic gynecological conditions including menstrual disorders, while lack of health insurance rendered some treatments unaffordable as they were only available on a fee-for-service basis, disrupting care continuity and ultimately undermined their capacity to work. Giselle’s (25 years old) account describes the psychological toll of this situation:

I have an ovarian cyst, and I had an appointment last week, but I couldn’t go because I didn’t have the money. I have menstrual disorders. Condoms irritate me. I have to buy lubricant. Obviously, that’s on me. And I’ll tell you, I can’t stand it. And now that I have a cyst, it scares me to death.

Additionally, for more complex conditions requiring expensive treatment, participants had no choice but to delay medical treatment until they were able to obtain SIS insurance. Maria Fernanda (33 years old), who required uterine fibroid surgery, explained that her only option was to wait an indeterminate time for her foreign resident card before she could begin the process of obtaining SIS insurance since the treatment remained unaffordable for her through the private system:

It has been difficult for me. I’m bleeding constantly, and the doctor told me that I need surgery, and with the PTP (Temporary Resident card), you can’t access the SIS. I have to wait on the foreigner’s card to be able to opt for the SIS. In a private clinic, the surgery is at least 4,000 Soles (~1,130USD), where do I get that money? So, I am waiting to see. I try not to go to work. I just offer company to clients, but I try not to do service. It is uncomfortable, apart from the pain, but that’s one’s sad life.

Participants further described how untreated conditions, such as pelvic pain, vaginal bleeding, and inflammation, became normalized features of daily life, reflecting the absence of accessible and responsive care. For some, these conditions remained unaddressed and so persistent that they were viewed as an unavoidable yet accepted part of their work. Such was the situation for Natalia (35 years old), who described: “Sometimes when I get my period, on the first day I don’t work because it’s really bad. The second day I have to wear a tampon, it bothers me, the discomfort and I get swollen, but I still have to work because I need money.” Similarly, Daniela’s (29 years old) narrative exemplifies how her internalized acceptance of pain and the physical toll of sex work have erased her sense of entitlement to care and reframed her agency solely around economic necessity and survival: “Some clients are rough and being with so many people one night it hurts you, but you have to keep working to make ends meet.”

### 3. Community care and solidarity among sex workers

In the context of structural exclusion from formal healthcare systems, and ongoing systemic inattention to SRH needs and persistent discrimination within the public healthcare system, participants described turning to one another and to community-based organizations as essential sources of self-care, information, and emotional support. For example, Elena (38 years old) shared: “I went to an organization where I was seen by a psychologist because at that time I was going through separation from my husband as I was being abused psychologically. The psychologist helped me a lot to overcome the situation”. These community-based systems of care emerged as both practical strategies for survival and as collective responses to institutional abandonment. Participants further emphasized the importance of peer-driven care practices, extending beyond condom use to meet their complex SRH needs, as Lucia (23 years old) noted:

“I once got tested for HIV and syphilis through a health outreach campaign for Venezuelans. But in health centers or hospitals, if you do not have SIS or foreigner’s card, you do not have healthcare because they do not take you into account. There, they prefer the person to die. If you don’t have money, you don’t have health. I was going through a [spontaneous] abortion and I went three times to the hospital and they did not assist me, because I did not have SIS, I do not have a foreigner’s card.”

Faced with the multiple experiences of institutional neglect and hostility while accessing SRH services, participants cultivated peer information-sharing networks focused on occupational self-care. These exchanges became a vital means of navigating health risks in the absence of reliable public health guidance. However, participants were also exposed to the spread of collective misinformation. As described by Lizbeth (28 years old): “I use vaginal suppositories and vinegar. I do it when I’m at home. Vinegar kills all those infections you have. I have friends who are also sex workers, and they are the ones who talk and I listen to and so on.”

Peer networks were also central to helping women access SRH services that would otherwise be out of reach. Participants described coordinating with other migrant sex workers to access condoms and navigate the health system by leveraging their status as sex workers: “The girls were the ones who took me. They told me that we are not going to buy condoms because they give us condoms at the *posta*. We say that we are sex workers and get seen that same day. We receive a box of 100 [condoms] that’s what you work with.” (Sofia, 39 years old).

CBOs and NGOs emerged as essential resources in the absence of accessible, non-discriminatory public health services. It was common for participants to describe these organizations as not only reliable sources of material and legal support but also as trusted providers of comprehensive SRH care, covering the costs of consultations, diagnostic procedures, hospitalizations, and medications, in contrast to public institutions, where care was often perceived as conditional, stigmatizing, or outright denied. Maria Fernanda (33 years old) described the support she received from one such organization:

“They helped me a lot with the payment system, at least with the ultrasounds, the hospitalization, they paid for all of that, because if you don’t have insurance, they don’t take care of you. I was hospitalized until the bleeding stopped, and they put me on treatment for that. They helped me a lot, they paid for my consultations, they paid for my treatment. There was a girl who was in charge of making the payments to the hospital, the payments for the medicines at the pharmacy and so on. They helped me a lot.”

These support systems were described not only as materially necessary but emotionally sustaining. Participants returned repeatedly to the idea that NGOs were always present, attentive, and prepared to respond in times of crisis, an experience that contrasted sharply with public health services. As Andrea (45 years old) shared, “You always go to the NGOs because they are always there to support you. The person in charge is always supporting you in any emergency, anything you need. She is always supporting you.”

## Discussion

Participants in this study described navigating a landscape of precarious, stigmatized, and highly conditional access to SRH care, limited to condom distribution and HIV/STI testing, while broader preventive and occupational SRH services remained unaddressed. Rather than isolated barriers, findings reflect how access to care is structured by intersecting forms of stigma tied to sex work and migration status, which shape both the availability and conditions of care. At the same time, participants turned to peer networks, NGOs and CBOs for emotional, material, and clinical assistance. While these networks provided critical support, some shared practices were not evidence-based (e.g., using vinegar to prevent vaginal infections), highlighting gaps in access to reliable, institutional SRH information and overall SRH services.

Guided by the Structural Vulnerability Framework, these findings illustrate how SRH access is patterned by participants’ positioning within intersecting social hierarchies, institutional arrangements, and economic systems that systematically constrain access to resources and care [40,41]. In the Peruvian context, these constrains are currently exemplified by reports of healthcare staff shortages and service disruptions in medical centers serving sex workers [49], and by documentation of abuse by local authorities and non-state actors [50]. Further, these structural conditions are reinforced by institutional policies (i.e., Ministry of Health) that prioritize individual-level HIV/STI surveillance, leaving occupational and preventive SRH needs unaddressed. Importantly, access to care is not only limited, but conditional, mediated through bureaucratic systems tied to migratory status and occupation, thereby reproducing exclusion despite formal guarantees of universal healthcare access [26–32].

Participants in this study demonstrated resourcefulness in response to structural gaps in healthcare. Their reliance on alternative channels, including NGOs, CBOs and peer networks, for health information, social support, and SRH services, reflects strategies well documented among marginalized populations navigating institutional exclusion [16,27]. These practices can be understood as forms of constrained agency, emerging within contexts where formal systems fail to meet basic health needs. Abu-Lughod [51] cautions against the tendency to celebrate such strategies as signs of emancipation and instead, render visible the ineffectiveness of institutional systems. While participants exercised agency, this agency was constrained and shaped by the very structures they sought to navigate. Their turn to private healthcare services and out-of-pocket payments, a pattern frequently documented among sex workers in Peru [16,23,44,52], illustrates this dynamic. Rather than simply reflecting choice or preference, these practices may also signal the normalization of structural exclusion, where out-of-pocket care is accepted as a necessary condition of survival rather than a failure of the public system.

Although participants actively build their own support systems, some shared information within these networks may be harmful rather than preventive (e.g., using vinegar to prevent vaginal infections), revealing a lack of accessible and reliable information about SRH safe practices [16,53]. This tension underscores a critical limitation of community-based care operating in isolation from formal systems, highlighting the need for integration rather than substitution, fostering stronger relationships between sex workers, healthcare providers, and institutional systems to implement models such as structural competency [54] for healthcare providers and institutions.

Addressing the multilevel and systemic exclusion documented in this study, structural reforms are urgently needed that move beyond narrow HIV/STI surveillance toward comprehensive SRH care. First, public health systems must integrate non-stigmatizing comprehensive SRH into HIV/STI programs while including occupation-specific care. For example, the Planned Parenthood Association of Thailand (PPAT), in collaboration with the Ministry of Health, offers sex workers no-cost or subsidized reproductive tract cancer screening with established pathways for referral to government hospitals for treatment [55]. Secondly, translating Peru’s legal guarantees into practice requires addressing bureaucratic barriers for migrants, including decoupling migratory status from care eligibility [16,27]. Third, supporting peer networks and community-based organizations should be prioritized, not as substitutes for state responsibility, but as complements to population-level impact. For example, partnerships between community-based organizations and primary care centers (i.e., *postas*) can integrate sex workers as peer providers to facilitate access to health information and foster mutual aid and emotional support. The ongoing rise of anti-NGOs laws in Peru [56,57] threatens the very organizations that sex workers in this study relied upon for accessible and dignified SRH services, underscoring the urgency of these reforms.

These reforms must be implemented to realize the promise of universal access detailed in Peru’s Sexual and Reproductive Health Law (2010) and the WHO’s Health For All principle. Comprehensive SRH access is not solely a biomedical issue, but a prerequisite for safe work and economic stability that should be embedded into a rights-based approach [27,44,50,58,59], premise that has been persistently remarked by global institutions such as the Committee on Economic, Social and Cultural Rights (CESCR), the Committee on the Elimination of Discrimination against Women (CEDAW), and the WHO [60,61], which clearly indicated that women’s right to health includes their SRH.

### Limitations

While this qualitative study provides nuanced insights into the experiences of Venezuelan women sex workers in Peru, its findings cannot be seen as fully representative of this population in Peru. An additional consideration is that this research was designed more broadly to explore the intersections between migration, sex work, and health among Venezuelan migrants in Peru belonging to populations at elevated risk of HIV and other STIs: men who have sex with men, transgender women, and cisgender women sex workers. During data collection and analysis, SRH emerged as a significant theme specifically among cisgender women participants, particularly regarding access to SRH services and self-protective practices against occupational health risks. Additionally, despite sex workers’ elevated risk of unintended pregnancies, we were unable to explore more in-depth abortion experiences due to legal restrictions in Peru (therapeutic abortion permitted only under life-threatening circumstances before 22 weeks of pregnancy).

## Conclusion

These qualitative findings from interviews with Venezuelan ciswomen who have migrated to Peru and engage in sex work suggest that access to SRH is shaped by the intersection of institutional policies, migration status, and stigmatized labour, which together produce patterned exclusion from healthcare systems beyond individual-level risk. Participants’ experiences of conditional care, reliance on out-of-pocket services, and limited access to comprehensive SRH highlight how structural conditions constrain both the availability and quality of care. Despite global and national commitments to universal health access, including those articulated by the WHO and Peruvian policy frameworks, a persistent gap between legislation and implementation continues to exclude Venezuelan sex workers from meaningful access to care and social protections. Addressing these inequities requires urgent approaches that center occupational health and migration-related vulnerabilities within broader efforts to advance SRH equity in Latin America and beyond. First, generate a legal framework that ensures access to public healthcare for migrants in Peru who do not have a foreigner’s ID and still rely on out-of-pocket spending due to lack of access to this human right. Second, HIV/STI programs must be expanded to incorporate comprehensive SRH services beyond testing and condom distribution, as part of the comprehensive approach to HIV prevention and treatment already established by Ministry of Health regulations. This integration can be achieved at the first level of care (i.e., primary healthcare centers) and by establishing mechanisms that allow for cost-free referrals between specialties or programs (e.g., HIV/STI programs and gynecological services). Finally, drawing from structural competency models, co-develop with peer networks and communities, programs aimed at eradicating stigma and discrimination by healthcare personnel in public facilities, improving access to services and providing accurate SRH information.

Without targeted policy reform and sustained investment in inclusive, non-stigmatizing health systems, efforts to expand SRH services risk reproducing the structural inequities they seek to address, limiting progress toward social equity and reproductive justice.
