# Supplementary material for: Navigating sexual and reproductive health among Venezuelan women sex workers in Peru: Qualitative explorations of structural vulnerability and resistance
Source: PLOS Glob Public Health. 2026 Jul 27;6(7):e0006906. doi: 10.1371/journal.pgph.0006906 (PMC13405076; doi:10.1371/journal.pgph.0006906)
Supplement: Attachment — Submitted filename: Response.docx [file pgph.0006906.s001.docx]

**Submission ID: PGHP-D-25-03790**

**Title:** Navigating sexual and reproductive health among Venezuelan women sex workers in Peru: Qualitative explorations of structural vulnerability and resistance.

**We sincerely thank the reviewers for their valuable and insightful feedback. We have carefully considered and incorporated their suggestions, which, we believe, have significantly strengthened the arguments and our paper overall.**

**Reviewer No. 1**

Thank you for this pertinent and powerful work, I’m grateful to have had the chance to read it. Appreciate your discussion of the tension between understanding participant agency and the wider structures that interact with them, and also your use of quotes to tell the story of these women. Congratulations!

**Thank you for your generous and encouraging feedback. I am pleased that the discussion of agency within structural constraints and the use of participants' voices resonated with you. Your thoughtful engagement with the work is much appreciated.**

Paper mentions the Structural Vulnerability Framework, but it isn’t really clear to me how you are using it to make sense of and represent your results/discussion. Would be helpful to have a bit more explicit discussion of this.

**Thank you for raising this point. We have added the following clarification of the framework in the introduction to clarify its application to the results and discussion sections:**

***The Structural Vulnerability Framework conceptualizes health outcomes as products of political, economic, and social arrangements rather than individual behaviors, emphasizing how intersecting systems of inequality, such as migration status and gender, produce patterned exposure to harm. It also serves as a practical lens for identifying and addressing these structural barriers through systems-level interventions***.

**Additionally, the following information was included in the Results section:**

***Drawing on the Structural Vulnerability Framework, participants’ experiences are analyzed and presented below as shaped by structural forces, including migration policy, labour precarity, and gendered stigma, that constrain access to care and produce pattern inequalities in SRH outcomes. The three interrelated themes identified include: 1. Conditional care shaped by structural factors, including institutional policies, migration status, and stigmatized forms of labour that structure availability and the terms under which SRH is accessed; 2. Occupational health access constrained by economic and bureaucratic contexts, shaped by out-of-pocket expenditures, insurance eligibility and institutional responses to stigmatized labour; and 3. Community-based care in response to structural exclusion and conditional access, exercising agency within structural constraints***

32-34 confusingly phrased sentence

**We appreciate the observation. For more clarity we have edit as:**

***Participants described access to SRH services in Peru as narrowly restricted to HIV and sexually transmitted infection (STI) testing and condom distribution, with accessibility to those services often contingent upon disclosing involvement in sex work, thereby reinforcing surveillance, stigma, and experiences of mistreatment in healthcare settings.***

66 ‘recognized as workers’ by who?

**Thank you for outlining this clarification. We have edited the sentence as**

**followed:**

***Within the public health system, sex workers are not formally recognized as workers, instead they are classified as a “high-risk group” and are primarily approached through a disease-prevention lens in relation to sexually transmitted infections (STIs), including HIV [23].***

80 it might be helpful to more explicitly reiterate in this paragraph how migrants are included within the various health laws described, so it’s clear how the migration component intersects with the sex worker one (at least on paper), i.e. why it’s important to study migrant sex workers specifically, rather than sex workers generally. As indicated in paragraph 1, their needs are nuanced.

**Thank you for the suggestion. We have added the following:**

***Taken together, these legal frameworks suggest that Venezuelan migrant sex workers should, at least on paper, be doubly protected: as migrants formally included within universal health guarantees and as individuals entitled to non-discriminatory SRH care irrespective of occupation. However, previous studies have shown that Venezuelan migrant women are subject to heightened discrimination in healthcare settings, including SRH care services [23,35,36]. Furthermore, while SIS eligibility has been extended to individuals up to 42 days postpartum regardless of migration status and health insurance [37,38], barriers persist for migrant sex workers who are unable to retain SIS eligibility.***

110 makes me think of the work of Lila Abu-Lughod on “the romance of resistance” and how agency interacts with structure in a complicated way, not always ‘fighting back’

**Thank you for this insightful suggestion. We have added Abu-Lughod’s reference in the discussion section:**

***These practices can be understood as forms of constrained agency, emerging within contexts where formal systems fail to meet basic health needs. Abu-Lughod [51] cautions against the tendency to celebrate such strategies as signs of emancipation and instead, render visible the ineffectiveness of institutional systems. While participants exercised agency, this agency was constrained and shaped by the very structures they sought to navigate. Their turn to private healthcare services and out-of-pocket payments, a pattern frequently documented among sex workers in Peru [16,23,44,52], illustrates this dynamic. Rather than simply reflecting choice or preference, these practices may also signal the normalization of structural exclusion, where out-of-pocket care is accepted as a necessary condition of survival rather than a failure of the public system.***

142 what kind of saturation? Is there anywhere you want to reflect on or highlight how interviews might draw out certain representations of the participants’ experiences, rather than the full picture?

**Thanks for this insightful recommendation. We have edited the paragraph for clarity as follows:**

***Sample size was determined by information power and supported by ongoing assessment of thematic sufficiency [46], which allowed for adjusting the sample based on the relevance of the obtained information and for identifying key patterns among participants as data collection progressed. Given the study’s focused objective on sexual and reproductive health (SRH) among Venezuelan migrant women engaged in sex work in Lima, the specificity of the sample, and the depth of the in-depth interviews, we determined that a sample of 34 participants provided sufficient informational richness to support robust analysis. This assessment was supported by iterative team discussions during data collection and preliminary analysis, which indicated that additional interviews were yielding limited novel insights relevant to the study objectives. Accordingly, the findings reflect participants situated experiences and interpretations rather than an exhaustive account of all dimensions of SRH access.***

149 maybe a sentence somewhere on why specifically cis women

**Thanks for your insight. We agree it is crucial to include this information. We have added the following paragraph within the Introduction and under Participants and data collection section:**

**Introduction:**

***Cisgender Venezuelan women in Peru are subjected to hypersexualized stereotypes and xenophobic attitudes [20,21], and face an even greater burden of chronic health conditions and unmet sexual and reproductive health (SRH) needs. For instance, only 20.6% of Venezuelan women received family planning services, compared to 56% of the general population in Peru [22]***

**Participants and data collection:**

***This study focused specifically on cisgender women, given the convergence of intersecting structural conditions shaping their engagement with sex work and access to SRH services in Peru and their specific sex- and gender-based SRH needs [44,45]***

176 would appreciate a statement on researcher positionality, which could help unpack how participants engaged with interviews

**Thank you for raising this point. We have added the following positionality statement at the end of the Data collection and participants’ section:**

***Interviews were conducted by three cisgender women researchers (two Peruvian and one Venezuelan) who had formal training in qualitative methods and prior experience working with sex worker communities. The decision to have cisgender women conduct the interviews was intentional, with the aim of fostering rapport and facilitating open discussion of highly sensitive topics related to sex work, migration, and sexual and reproductive health. At the same time, we recognize that shared gender identity does not eliminate power differentials, and that researchers’ social positionalities may have shaped both what participants chose to disclose and how experiences were narrated. We remained attentive to how cultural and national origin, as well as institutional affiliation, may have influenced participants’ expectations of the research and the ways in which they framed their narratives. All interviews were conducted in private settings to promote confidentiality and participant comfort. Prior to each interview, researchers emphasized the voluntary nature of participation, the confidentiality of responses, and the study’s purpose of creating space for participants to share their perspectives, with the broader goal of informing advocacy and policy measures responsive to their needs.***

203 thank you for this poignant quote. I’m wondering if there is another line from this interview where it’s clear that the treatment Valeria experiences is linked to her migration/sex worker status rather than other potential vulnerabilities/biases? This comes up a couple of times in the results e.g. lines 309-312, where it isn’t necessarily clear whether this is a problem specific to migrant cis women who are sex workers, versus related to e.g. poverty. Maybe it doesn’t have to be specific to them, in which case could mention that too.

**We appreciate your insightful observation to elaborate on this quote. We have added this sentence from participant’s interview in line 203 from Valeria:**

***Most of them [doctors] see us as weirdos, first because we are Venezuelan, and if they know about the sex work, they treat you even worse.***

**And in line 309-312 reflects neglect contingent upon the participant’s migration status*:***

***I was going through an [spontaneous] abortion and I went three times to the hospital and they did not assist me, because I did not have SIS, I do not have foreigner’s card ”***

299 subheading 3!

**Thank you for pointing this out. We have corrected the subheading accordingly.**

**Reviewer No. 2**

Abstract: The abstract clearly summarizes the context, design, key findings, and implications, but it would benefit from a clearer statement of novelty and analytic contribution.

**Thanks for this suggestion. We have edited the abstract to integrate a clearer statement of novelty and analytic contribution while staying within the word limit.**

Introduction: The introduction provides strong legal and structural background; however, it is dense and could better synthesize prior empirical literature while sharpening the research gap and objectives earlier.

**Thank you for this insight. We have edited the introduction by removing some sections and synthesized prior empirical literature while sharpening the research gap:**

***However, previous studies have shown that Venezuelan migrant women are subject to heightened discrimination in healthcare settings [23,35,36].***

**We also state the study’s objective earlier at the end of the first paragraph of the introduction:**

***Against this complex backdrop, this study examines how Venezuelan migrant cisgender women engaged in sex work in Lima navigate SRH services, with attention to how migration, gender, and labour intersect to shape SRH vulnerabilities and access to care.***

Methods: The qualitative design and analytic framework are appropriate and generally well described. Still, greater detail on recruitment processes, researcher reflexivity, saturation determination, and ethical safeguards would improve methodological transparency and rigor

**Thanks for this suggestion. We have developed the following methodological elements for each of the respective sections:**

**Recruitment:**

***Recruitment was conducted in collaboration with ‘Asociación de Trabajadoras Sexuales Miluska Vida y Dignidad’, a community-based organization (CBO) with a strong presence in Peru that advocates for the social inclusion and health rights of sex workers. CBO members, which included Peruvian and Venezuelan migrant cisgender women sex workers, distributed flyers with study information in settings where sex workers were present and leveraged pre-existing connections between the organization and potential participants. Additionally, enrolled participants circulated information about the study and invited other eligible Venezuelan women residing in Lima through snowball sampling. This study focused specifically on cisgender women, given the convergence of intersecting structural conditions shaping their engagement with sex work and access to SRH services in Peru and their specific sex- and gender-based SRH needs [44,45]***

***Sample size was determined by information power and supported by ongoing assessment of thematic sufficiency [46], which allowed for adjusting the sample based on the relevance of the obtained information and for identifying key patterns among participants as data collection progressed. Given the study’s focused objective on sexual and reproductive health (SRH) among Venezuelan migrant women engaged in sex work in Lima, the specificity of the sample, and the depth of the in-depth interviews, we determined that a sample of 34 participants provided sufficient informational richness to support robust analysis. This assessment was supported by iterative team discussions during data collection and preliminary analysis, which indicated that additional interviews were yielding limited novel insights relevant to the study objectives. Accordingly, the findings reflect participants situated experiences and interpretations rather than an exhaustive account of all dimensions of SRH access. As a data collection method, the team developed a semi-structured interview guide that touched on the following core themes: exploring migration trajectories, SRH needs during relocation, perceptions of sex work, and HIV/STI care. The research team, comprised of native Spanish-speaking qualitative researchers, conducted in-person interviews lasting approximately 60 to 90 minutes in a private space.***

**Researcher reflexivity:**

***Interviews were conducted by three cisgender women researchers (two Peruvian and one Venezuelan) who had formal training in qualitative methods and prior experience working with sex worker communities. The decision to have cisgender women conduct the interviews was intentional, with the aim of fostering rapport and facilitating open discussion of highly sensitive topics related to sex work, migration, and sexual and reproductive health. At the same time, we recognize that shared gender identity does not eliminate power differentials, and that researchers’ social positionalities may have shaped both what participants chose to disclose and how experiences were narrated. We remained attentive to how cultural and national origins, as well as institutional affiliation, may have influenced participants’ expectations of the research and the ways in which they framed their narratives. All interviews were conducted in private settings to promote confidentiality and participant comfort. Prior to each interview, researchers emphasized the voluntary nature of participation, the confidentiality of responses, and the study’s purpose of creating space for participants to share their perspectives, with the broader goal of informing advocacy and policy measures responsive to their needs.***

**Ethical safeguards:**

***Participants were assigned pseudonyms to protect their identities. Audio recordings and transcripts were uploaded to a password-protected server accessible only to select team members. As per institutional policies, the data will be retained for 7 years after data collection is completed, then destroyed.***

Results: Findings are rich and well supported by participant quotations, effectively illustrating stigma, conditional care, and reliance on peer and community networks.

**We sincerely appreciate your encouraging and insightful comment, as well as your recognition of the importance of this work.**

Conclusions: More specific actionable recommendations would enhance the manuscript’s overall impact.

**Thank you for this suggestion. We have included the following actionable and specific recommendations within the conclusion:**

***These qualitative findings from interviews with Venezuelan ciswomen who have migrated to Peru and engage in sex work suggest that access to SRH is shaped by the intersection of institutional policies, migration status, and stigmatized labour, which together produce patterned exclusion from healthcare systems beyond individual-level risk. Participants’ experiences of conditional care, reliance on out-of-pocket services, and limited access to comprehensive SRH highlight how structural conditions constrain both the availability and quality of care. Despite global and national commitments to universal health access, including those articulated by the WHO and Peruvian policy frameworks, a persistent gap between legislation and implementation continues to exclude Venezuelan sex workers from meaningful access to care and social protections. Addressing these inequities requires urgent approaches that center occupational health and migration-related vulnerabilities within broader efforts to advance SRH equity in Latin America and beyond. First, generate a legal framework that ensures access to public healthcare for migrants in Peru who do not have a foreigner's ID and still rely on out-of-pocket spending due to lack of access to this human right. Second, HIV/STI programs must be expanded to incorporate comprehensive SRH services beyond testing and condom distribution, as part of the comprehensive approach to HIV prevention and treatment already established by Ministry of Health regulations. This integration can be achieved at the first level of care (i.e., primary healthcare centers) and by establishing mechanisms that allow for cost-free referrals between specialties or programs (e.g., HIV/STI programs and gynecological services). Finally, drawing from structural competency models, co-develop with peer networks and communities, programs aimed at eradicating stigma and discrimination by healthcare personnel in public facilities, improving access to services and providing accurate SRH information. Without targeted policy reform and sustained investment in inclusive, non-stigmatizing health systems, efforts to expand SRH services risk reproducing the structural inequities they seek to address, limiting progress toward social equity and reproductive justice.***
