# Supplementary material for: Navigating sexual and reproductive health among Venezuelan women sex workers in Peru: Qualitative explorations of structural vulnerability and resistance
Source: PLOS Glob Public Health. 2026 Jul 27;6(7):e0006906. doi: 10.1371/journal.pgph.0006906 (PMC13405076; doi:10.1371/journal.pgph.0006906)
Supplement: Peer review history

# Peer review: Decision Letter 0

**DOI:** 10.1371/journal.pgph.0006906.r001

**Authors:** Dumile Gumede

19 Feb 2026

PGPH-D-25-03790

Navigating sexual and reproductive health among Venezuelan women sex workers in Peru: Qualitative explorations of structural vulnerability and resistance.

PLOS Global Public Health

Dear Dr. Castro-Arteaga,

Thank you for submitting your manuscript to PLOS Global Public Health. After careful consideration, we feel that it has merit but does not fully meet PLOS Global Public Health’s publication criteria as it currently stands. Therefore, we invite you to submit a revised version of the manuscript that addresses the points raised during the review process.

Please submit your revised manuscript by Apr 05 2026 11:59PM. If you will need more time than this to complete your revisions, please reply to this message or contact the journal office at globalpubhealth@plos.org. When you're ready to submit your revision, log on to https://www.editorialmanager.com/pgph/ and select the 'Submissions Needing Revision' folder to locate your manuscript file.

Please include the following items when submitting your revised manuscript:

- A letter that responds to each point raised by the editor and reviewer(s). You should upload this letter as a separate file labeled 'Response to Reviewers'.
- A marked-up copy of your manuscript that highlights changes made to the original version. You should upload this as a separate file labeled 'Revised Manuscript with Track Changes'.
- An unmarked version of your revised paper without tracked changes. You should upload this as a separate file labeled 'Manuscript'.

Guidelines for resubmitting your figure files are available below the reviewer comments at the end of this letter.

We look forward to receiving your revised manuscript.

Kind regards,

Dumile Gumede, PhD (Health Promotion)

Academic Editor

PLOS Global Public Health

Journal Requirements:

1. Please include a complete copy of PLOS’ questionnaire on inclusivity in global research in your revised manuscript. Our policy for research in this area aims to improve transparency in the reporting of research performed outside of researchers’ own country or community. The policy applies to researchers who have travelled to a different country to conduct research, research with Indigenous populations or their lands, and research on cultural artefacts. The questionnaire can also be requested at the journal’s discretion for any other submissions, even if these conditions are not met.  Please find more information on the policy and a link to download a blank copy of the questionnaire here: https://journals.plos.org/globalpublichealth/s/best-practices-in-research-reporting. Please upload a completed version of your questionnaire as Supporting Information when you resubmit your manuscript.

2. Please amend your detailed Financial Disclosure statement. This is published with the article. It must therefore be completed in full sentences and contain the exact wording you wish to be published.

i. Please clarify all sources of financial support for your study. List the grants, grant numbers, and organizations that funded your study, including funding received from your institution. Please note that suppliers of material support, including research materials, should be recognized in the Acknowledgements section rather than in the Financial Disclosure.

ii. State the initials, alongside each funding source, of each author to receive each grant. For example: "This work was supported by the National Institutes of Health (####### to AM; ###### to CJ) and the National Science Foundation (###### to AM)."

iii. State what role the funders took in the study. If the funders had no role in your study, please state: “The funders had no role in study design, data collection and analysis, decision to publish, or preparation of the manuscript.”

iv. If any authors received a salary from any of your funders, please state which authors and which funders.

3. Please ensure that your Ethics Statement is available in its entirety at the beginning of your Methods section, under a subheading 'Ethics Statement'.

4. In the online submission form, you indicated that “The data that support the findings of this study are available from the corresponding author upon reasonable request.”.

All PLOS journals now require all data underlying the findings described in their manuscript to be freely available to other researchers, either

1. In a public repository,

2. Within the manuscript itself, or

3. Uploaded as supplementary information.

This policy applies to all data except where public deposition would breach compliance with the protocol approved by your research ethics board. If your data cannot be made publicly available for ethical or legal reasons (e.g., public availability would compromise patient privacy), please explain your reasons by return email and your exemption request will be escalated to the editor for approval. Your exemption request will be handled independently and will not hold up the peer review process, but will need to be resolved should your manuscript be accepted for publication. One of the Editorial team will then be in touch if there are any issues.

If the reviewer comments include a recommendation to cite specific previously published works, please review and evaluate these publications to determine whether they are relevant and should be cited. There is no requirement to cite these works unless the editor has indicated otherwise.

Additional Editor Comments (if provided):

[Note: HTML markup is below. Please do not edit.]

Reviewers' comments:

Reviewer's Responses to Questions

**Comments to the Author**

1. Does this manuscript meet PLOS Global Public Health’s publication criteria? Is the manuscript technically sound, and do the data support the conclusions? The manuscript must describe methodologically and ethically rigorous research with conclusions that are appropriately drawn based on the data presented.

Reviewer #1: Yes

Reviewer #2: Partly

**********

2. Has the statistical analysis been performed appropriately and rigorously?

Reviewer #1: N/A

Reviewer #2: N/A

**********

3. Have the authors made all data underlying the findings in their manuscript fully available (please refer to the Data Availability Statement at the start of the manuscript PDF file)?

The PLOS Data policy requires authors to make all data underlying the findings described in their manuscript fully available without restriction, with rare exception. The data should be provided as part of the manuscript or its supporting information, or deposited to a public repository. For example, in addition to summary statistics, the data points behind means, medians and variance measures should be available. If there are restrictions on publicly sharing data—e.g. participant privacy or use of data from a third party—those must be specified.

Reviewer #1: Yes

Reviewer #2: No

**********

4. Is the manuscript presented in an intelligible fashion and written in standard English?

PLOS Global Public Health does not copyedit accepted manuscripts, so the language in submitted articles must be clear, correct, and unambiguous. Any typographical or grammatical errors should be corrected at revision, so please note any specific errors here.

Reviewer #1: Yes

Reviewer #2: No

**********

5. Review Comments to the Author

Please use the space provided to explain your answers to the questions above. You may also include additional comments for the author, including concerns about dual publication, research ethics, or publication ethics. (Please upload your review as an attachment if it exceeds 20,000 characters)

Reviewer #1: Thank you for this pertinent and powerful work, I’m grateful to have had the chance to read it. Appreciate your discussion of the tension between understanding participant agency and the wider structures that interact with them, and also your use of quotes to tell the story of these women. Congratulations!

A couple of small suggestions below and one general one here: Paper mentions the Structural Vulnerability Framework, but it isn’t really clear to me how you are using it to make sense of and represent your results/discussion. Would be helpful to have a bit more explicit discussion of this.

Lines

32-34 confusingly phrased sentence

66 ‘recognized as workers’ by who?

80 it might be helpful to more explicitly reiterate in this paragraph how migrants are included within the various health laws described, so it’s clear how the migration component intersects with the sex worker one (at least on paper), i.e. why it’s important to study migrant sex workers specifically, rather than sex workers generally. As indicated in paragraph 1, their needs are nuanced.

110 makes me think of the work of Lila Abu-Lughod on “the romance of resistance” and how agency interacts with structure in a complicated way, not always ‘fighting back’…

142 what kind of saturation? Is there anywhere you want to reflect on or highlight how interviews might draw out certain representations of the participants’ experiences, rather than the full picture?

149 maybe a sentence somewhere on why specifically cis women

176 would appreciate a statement on researcher positionality, which could help unpack how participants engaged with interviews

203 thank you for this poignant quote. I’m wondering if there is another line from this interview where it’s clear that the treatment Valeria experiences is linked to her migration/sex worker status rather than other potential vulnerabilities/biases? This comes up a couple of times in the results e.g. lines 309-312, where it isn’t necessarily clear whether this is a problem specific to migrant cis women who are sex workers, versus related to e.g. poverty. Maybe it doesn’t have to be specific to them, in which case could mention that too.

299 subheading 3!

Reviewer #2: Abstract: The abstract clearly summarizes the context, design, key findings, and implications, but it would benefit from a clearer statement of novelty and analytic contribution.

Introduction: The introduction provides strong legal and structural background; however, it is dense and could better synthesize prior empirical literature while sharpening the research gap and objectives earlier.

Methods: The qualitative design and analytic framework are appropriate and generally well described. Still, greater detail on recruitment processes, researcher reflexivity, saturation determination, and ethical safeguards would improve methodological transparency and rigor.

Results: Findings are rich and well supported by participant quotations, effectively illustrating stigma, conditional care, and reliance on peer and community networks.

Discussion: The discussion links results to structural vulnerability and policy relevance, though clearer separation between interpretation and evidence, reduced thematic overlap, and engagement with contrasting literature would strengthen analytical depth.

Conclusions: More specific actionable recommendations would enhance the manuscript’s overall impact.

**********

6. PLOS authors have the option to publish the peer review history of their article (what does this mean?). If published, this will include your full peer review and any attached files.

**Do you want your identity to be public for this peer review?**  If you choose “no”, your identity will remain anonymous but your review may still be made public.

For information about this choice, including consent withdrawal, please see our Privacy Policy.

Reviewer #1: No

Reviewer #2: **Yes:**  Abu Sayeed Md Abdullah,

Deputy Director, Reproductive and Child Health, CIPRB;

PhD Candidate, Department of Public Health Science, Mid Sweden University (MIUN), Sweden.

I am a dedicated and curious learner with a strong interest in public health specially maternal health. I approach tasks with diligence and attention to detail, always striving for excellence. I value continuous learning and actively seek opportunities to expand my knowledge and skills, aiming to make meaningful contributions in both my academic and professional pursuits.

**********

[NOTE: If reviewer comments were submitted as an attachment file, they will be attached to this email and accessible via the submission site. Please log into your account, locate the manuscript record, and check for the action link "View Attachments". If this link does not appear, there are no attachment files.]

-->Figure Resubmissions:

While revising your submission, we strongly recommend that you use PLOS’s NAAS tool (https://ngplosjournals.pagemajik.ai/artanalysis) to test your figure files. NAAS can convert your figure files to the TIFF file type and meet basic requirements (such as print size, resolution), or provide you with a report on issues that do not meet our requirements and that NAAS cannot fix.

After uploading your figures to PLOS’s NAAS tool - https://ngplosjournals.pagemajik.ai/artanalysis, NAAS will process the files provided and display the results in the "Uploaded Files" section of the page as the processing is complete. If the uploaded figures meet our requirements (or NAAS is able to fix the files to meet our requirements), the figure will be marked as "fixed" above. If NAAS is unable to fix the files, a red "failed" label will appear above. When NAAS has confirmed that the figure files meet our requirements, please download the file via the download option, and include these NAAS processed figure files when submitting your revised manuscript.

# Author comment: Author response to Decision Letter 1

**DOI:** 10.1371/journal.pgph.0006906.r002

16 Apr 2026

Attachment Submitted filename: Response.docx

# Editor report: Decision Letter 1

**DOI:** 10.1371/journal.pgph.0006906.r003

**Authors:** Julia Robinson

1 Jul 2026

Navigating sexual and reproductive health among Venezuelan women sex workers in Peru: Qualitative explorations of structural vulnerability and resistance.

PGPH-D-25-03790R1

Dear MD, MSc Castro-Arteaga,

We are pleased to inform you that your manuscript 'Navigating sexual and reproductive health among Venezuelan women sex workers in Peru: Qualitative explorations of structural vulnerability and resistance.' has been provisionally accepted for publication in PLOS Global Public Health.

Before your manuscript can be formally accepted you will need to complete some formatting changes, which you will receive in a follow up email. A member of our team will be in touch with a set of requests.

Please note that your manuscript will not be scheduled for publication until you have made the required changes, so a swift response is appreciated.

IMPORTANT: The editorial review process is now complete. PLOS will only permit corrections to spelling, formatting or significant scientific errors from this point onwards. Requests for major changes, or any which affect the scientific understanding of your work, will cause delays to the publication date of your manuscript.

If your institution or institutions have a press office, please notify them about your upcoming paper to help maximize its impact. If they'll be preparing press materials, please inform our press team as soon as possible -- no later than 48 hours after receiving the formal acceptance. Your manuscript will remain under strict press embargo until 2 pm Eastern Time on the date of publication. For more information, please contact globalpubhealth@plos.org.

Thank you again for supporting Open Access publishing; we are looking forward to publishing your work in PLOS Global Public Health.

Best regards,

Julia Robinson

Executive Editor

PLOS Global Public Health

***********************************************************

Reviewer Comments (if any, and for reference):
